# Proton versus photon radiotherapy for primary hepatocellular carcinoma: a propensity-matched analysis

**DOI:** 10.1186/s13014-020-01605-4

**Published:** 2020-06-30

**Authors:** Jen-Yu Cheng, Chieh-Min Liu, Yu-Ming Wang, Hsuan-Chih Hsu, Eng-Yen Huang, Tzu-Ting Huang, Ching-Hsin Lee, Sheng-Ping Hung, Bing-Shen Huang

**Affiliations:** 1 Department of Radiation Oncology, Kaohsiung Chang Gung Memorial Hospital, Chang Gung University College of Medicine, Kaohsiung, Taiwan; 2 School of Traditional Chinese Medicine, Chang Gung University, Taoyuan, Taiwan; 3 Department of Radiation Oncology, Chang Gung Memorial Hospital, Chang Gung University, Taoyuan, Taiwan; 4 Graduate Institute of Clinical Medicine, Chang Gung University, Taoyuan, Taiwan; 5 Department of Medical Imaging and Radiological Sciences, Chang Gung University, Taoyuan, Taiwan

**Keywords:** Hepatocellular carcinoma, Proton, Photon, Radiotherapy

## Abstract

**Background:**

Proton radiotherapy has a dosimetric advantage over photon radiotherapy. Many retrospective studies have shown promising results with proton radiotherapy in treating hepatocellular carcinoma (HCC). However, clinical evidence demonstrating the benefit of protons over photons is still limited. We therefore compared the clinical outcomes of the two modalities using medical research databases from our medical foundation.

**Methods:**

We conducted a propensity score-matched cohort study based on our multi-institution medical organization research database. From January 2007 to January 2018, a total of 413 patients (photon: 349; proton: 64) who were diagnosed with HCC and primarily treated with radiotherapy with curative intent were enrolled. Overall survival (OS) and radiation-induced liver disease (RILD) were assessed. Stratified analysis was also performed to evaluate the heterogeneous effects of the two arms.

**Results:**

A total of 110 patients (photon: 55; proton: 55) were analyzed in the propensity-matched series. The matched groups were balanced for baseline tumor risk factors. Cox regression analysis revealed a significant survival benefit in the proton group (*p* = 0.032, HR 0.56, 95% CI 0.33–0.96). The median overall survival in the proton group was not reached and that in the photon group was 17.4 months. The biological equivalent dose of radiotherapy was significantly higher in the proton group than in the photon group (median, 96.56 Gray [relative biological effectiveness] vs. 62.5 Gray, *p* < 0.001). The risk of RILD was significantly lower in the proton group (11.8% vs. 36%, *p* = 0.004).

**Conclusions:**

Proton radiotherapy could deliver a higher radiation dose than photon radiotherapy without increasing the risk of RILD and result in a better overall survival rate for those diagnosed with HCC and treated with radiotherapy with curative intent.

## Background

Radiotherapy is one of the crucial local treatment modalities for hepatocellular carcinoma (HCC). The delivery of a higher radiation dose to the target has been proven to result in better clinical outcomes [1–3]. However, the maximally tolerated dose for liver tumors is limited by not only the surrounding radiosensitive liver parenchyma but also the critical organs adjacent to the tumor target [4]. Compared to conventional photon radiotherapy techniques, proton radiotherapy offers dosimetric advantages because of its superior physical properties. A proton beam has a finite range of energy deposition with no exit dose after the target. This physical advantage over photon beams could therefore reduce unwanted spreading of the dose to the surrounding normal liver and adjacent organs [5]. Retrospective data from eastern and western countries have shown promising clinical results for proton radiotherapy [6, 7]. The three-year local control rate ranges from 70 to 95% depending on the tumor and the patients’ baseline characteristics, and the risk of toxicity is quite low.

There has only been one single-institution retrospective study, from the Massachusetts General Hospital (MGH), comparing the clinical benefits of proton radiotherapy over photon radiotherapy [8]. The study demonstrates survival benefits with proton therapy, which may be driven by a decreased incidence of radiation-related liver decompensation. However, the baseline characteristics of the two groups were not well balanced in the study. Patients in the proton radiotherapy group tended to have lower median Child-Pugh scores and better median albumin-bilirubin (ALBI) scores, which may raise concern regarding selection bias. Therefore, in this study, we investigated and compared the clinical outcomes of the two modalities using medical research databases from our multi-institution medical foundation with propensity score matching (PSM).

## Methods

### Data source

We conducted a retrospective analysis using deidentified data retrieved from the Chang Gung Research Database (CGRD). It is an electronic health record dataset derived from the Chang Gung Medical Foundation, which consists of a group of medical centers, regional hospitals, and local hospitals and provides approximately 13–14% of the cancer healthcare services in Taiwan [9]. Among them, two medical centers and two regional hospitals provide photon radiotherapy services. Cyclotron-based proton radiotherapy was available at one of the medical centers during the study period. This study was approved by the institutional review board (IRB) of the Chang Gung Medical Foundation (reference number 201901651B0).

### Study population

**Fig. 1:**
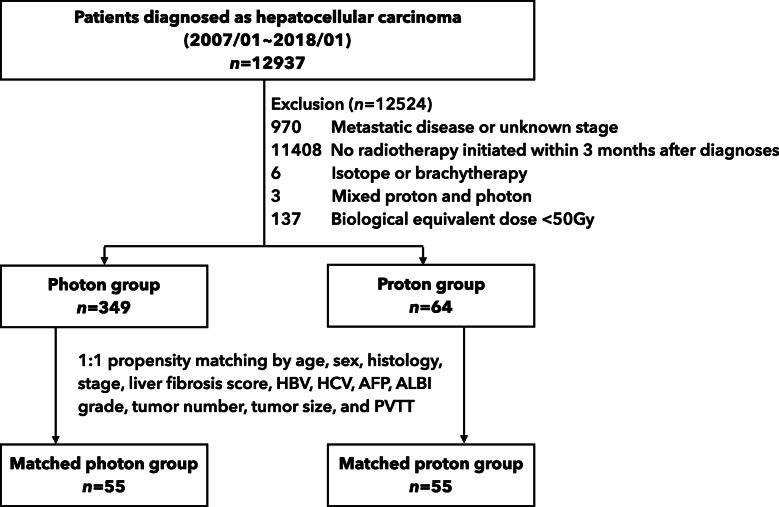
Study flowchart. Legend: Gy: Gray; HBV: hepatitis B; HCV: hepatitis C; AFP: alpha-fetoprotein; ALBI: albumin-bilirubin; PVTT: portal vein tumor thrombosis

Patients diagnosed with HCC and receiving local radiotherapy as a primary curative treatment modality between January 2007 and January 2018 were included in the study. To exclude patients treated with palliative intent, we excluded patients with an unknown stage, distant metastasis, or a biological equivalent dose (BED) below 50 Gray (Gy). The BED was calculated using an α/β value of 10 Gy. For proton radiotherapy, a relative biological effectiveness (RBE) value of 1.1 was used. The type of radiotherapy was further identified to group patients receiving photon and proton radiotherapy. Patients whose radiotherapy was not initiated within 3 months after diagnosis, received mixed photon and proton radiotherapy, or received isotope or brachytherapy were also excluded (Fig. 1).

### Covariates

Baseline variables considered in the analyses included patient age, sex, clinical American Joint Committee on Cancer (AJCC) stage, liver fibrosis score (Ishak scale), tumor differentiation, hepatitis B status, hepatitis C status, alpha-fetoprotein (AFP) status, ALBI grade, Child-Pugh class, tumor number, size of the largest tumor, and portal vein tumor thrombosis (PVTT) status. AJCC stage data were originally coded according to the 6th, 7th, or 8th edition depending on the year of the diagnosis and were all transformed to be in accordance with the 8th edition for the analysis.

### Outcomes

The primary outcome of this study was the overall survival (OS) of patients who were diagnosed with HCC and treated primarily with proton radiotherapy or photon radiotherapy with curative intent. OS was defined from the date of diagnosis to the date of death as a result of any cause. Radiation-induced liver disease (RILD) was also assessed to determine the toxicity of the treatment. There are two types of RILD. A patient who presented with anicteric hepatomegaly, ascites, and elevated alkaline phosphatase (more than twice the upper limit of normal or baseline value) 2 weeks to 3 months after therapy was defined as classic RILD positive. A patient whose liver transaminase levels were elevated more than five times the upper limit of normal (or more than twenty times the upper limit of normal in patients with baseline values more than five times the upper limit of normal) or whose Child-Pugh score worsened by two or more within 3 months after the completion of radiotherapy was defined as nonclassic RILD positive [10].

### Statistical analysis

PSM was applied to reduce selection bias between the study groups. Sex, age, clinical AJCC stage, liver fibrosis score, tumor differentiation, hepatitis B status, hepatitis C status, AFP status, ALBI grade, Child-Pugh class, tumor number, size of the largest tumor, and PVTT status were selected as independent variables. Using NCSS 10 Statistical Software (LLC, Kaysville, Utah, USA), the greedy method was used for matching at a 1:1 ratio between the study groups with a caliper width 0.2-fold the standard deviation of the propensity score between the study groups. The standardized mean difference (SMD) was used to evaluate covariate balance after PSM.

To compare the groups, we used the Pearson chi-square test or Fisher’s exact test for categorical variables and the Student t-test or Mann-Whitney test for continuous variables depending on the result of the Kolmogorov-Smirnov test for normality. OS was assessed using the Kaplan-Meier method and the Cox regression model. Stratified analyses for OS were performed to assess the heterogeneous effects of proton and photon radiotherapy. A two-sided *p*-value of < 0.05 was considered statistically significant. All statistical analyses were performed using SPSS statics v 25.0 (IBM Corp, Armonk, NY, USA) unless otherwise noted.

## Results

### Patient characteristics

A total of 12,937 patients diagnosed with HCC were identified. A total of 413 patients met the inclusion criteria and were analyzed (photon: 349 patients, proton: 64 patients) (Fig. 1). Compared with patients who received photon radiotherapy, at the patient level, those treated with proton radiotherapy tended to be older (mean age, 65.53 years vs. 60.38 years, *p* = 0.002), with more cases of hepatitis B or C infection (59.4% vs. 47.6% for hepatitis B, *p* = 0.001; 31.2% vs. 23.1% for hepatitis C, *P* = 0.001) and a higher liver fibrosis score (75.0% vs. 66.2% for Ishak F5–6) but better liver function (92.2% vs. 58.5% for Child-Pugh class A, *p* < 0.001; 37.5% vs 22.9% for ALBI grade 1, *p* < 0.001). At the tumor level, those treated with proton radiotherapy had an earlier clinical AJCC stage (40.6% vs. 9.2% for stage I/II and 31.6% vs. 79.9% for stage III, *p* < 0.001), lower AFP level (54.7% vs. 36.4% for AFP < = 200 ng/mL, *p* < 0.001), and fewer cases of PVTT (37.5% vs. 78.8%, *p* < 0.001).

After 1:1 PSM, 110 patients were analyzed (photon: 55 patients, proton: 55 patients). The baseline characteristics were balanced in the matched groups (SMD < 0.2 and *p* > 0.05 for all variables). Patient characteristics before and after matching are presented in Table 1.

**Table 1:** Patient characteristics before and after matching

Covariates	Before matching				After matching			
	Photon *n* = 349	Proton *n* = 64	*p*	SMD	Photon *n* = 55	Proton *n* = 55	*p*	SMD
Age, years (mean (SD))	60.38 (11.91)	65.53 (13.29)	0.002	0.408	62.27 (11.79)	63.90 (13.14)	0.495	0.131
Sex (%)			0.024	0.287			0.303	0.198
Male	292 (83.7)	46 (71.9)			48 (87.3)	44 (80.0)		
Female	57 (16.3)	18 (28.1)			7 (12.7)	11 (20.0)		
Histology (%)			0.867	0.207			1.000	0.067
Well differentiated	7 (2)	0 (0)			0 (0.0)	0 (0.0)		
Moderately differentiated	58 (16.6)	10 (15.6)			7 (12.7)	7 (12.7)		
Poorly differentiated	24 (6.9)	5 (7.8)			4 (7.3)	5 (9.1)		
Not available	260 (74.5)	49 (76.6)			44 (80.0)	43 (78.2)		
AJCC stage (%)			< 0.001	0.782			0.913	0.081
I/II	32 (9.2)	26 (40.6)			18 (32.7)	17 (30.9)		
III	279 (79.9)	33 (51.6)			31 (56.4)	33 (60.0)		
IVA	38 (10.9)	5 (7.8)			6 (10.9)	5 (9.1)		
Liver Fibrosis Score (%)			< 0.001	0.712			0.829	0.041
Ishak F1–4	51 (14.6)	16 (25.0)			14 (25.5)	15 (27.3)		
Ishak F5–6	231 (66.2)	48 (75.0)			41 (74.5)	40 (72.7)		
Not available	67 (19.2)	0 (0)			0 (0.0)	0 (0.0)		
Hepatitis B (%)			0.001	0.663			0.920	0.077
Negative	107 (30.7)	25 (39.1)			18 (32.7)	20 (36.4)		
Positive	166 (47.6)	38 (59.4)			36 (65.5)	34 (61.8)		
Missing	76 (21.8)	1 (1.6)			1 (1.8)	1 (1.8)		
Hepatitis C (%)			0.001	0.633			0.914	0.082
Negative	197 (56.4)	43 (67.2)			40 (72.7)	38 (69.1)		
Positive	81 (23.2)	20 (31.2)			14 (25.5)	16 (29.1)		
Missing	71 (20.3)	1 (1.6)			1 (1.8)	1 (1.8)		
Alpha-Fetoprotein (%)			< 0.001	0.719			0.701	0.073
< =200 ng/mL	127 (36.4)	35 (54.7)			30 (54.5)	32 (58.2)		
> 200 ng/mL	155 (44.4)	29 (45.3)			25 (45.5)	23 (41.8)		
Missing	67 (19.2)	0 (0)			0 (0)	0 (0)		
ALBI Grade (%)			< 0.001	0.610			0.604	0.192
1	80 (22.9)	24 (37.5)			21 (38.2)	20 (36.4)		
2	231 (66.2)	24 (37.5)			26 (47.3)	23 (41.8)		
Missing	38 (10.9)	16 (25.0)			8 (14.5)	12 (21.8)		
Child-Pugh class (%)			< 0.001	0.876			0.757	0.193
A	204 (58.5)	59 (92.2)			51 (92.7)	51 (92.7)		
B	68 (19.5)	4 (6.2)			2 (3.6)	4 (7.3)		
C	4 (1.1)	0 (0.0)			0 (0.0)	0 (0.0)		
Missing	73 (20.9)	1 (1.6)			2 (3.6)	1 (1.8)		
Tumor number (%)			0.157	0.229			0.741	0.198
Single	128 (36.7)	26 (40.6)			21 (38.2)	21 (38.2)		
Multiple	213 (61.0)	34 (53.1)			33 (60.0)	31 (56.4)		
Missing	8 (2.3)	4 (6.2)			1 (1.8)	3 (5.5)		
Size of the largest tumor (%)			0.270	0.305			0.809	0.125
< =5 cm	97 (27.8)	24 (37.5)			19 (34.5)	17 (30.9)		
> 5 and < =10 cm	132 (37.8)	23 (35.9)			22 (40.0)	21 (38.2)		
> 10 cm	111 (31.8)	17 (26.6)			14 (25.5)	17 (30.9)		
Missing	9 (2.6)	0 (0.0)			0 (0.0)	0 (0.0)		
PVTT (%)			< 0.001	0.922			0.669	0.199
Absent	66 (18.9)	36 (56.2)			28 (50.9)	28 (50.9)		
Present	275 (78.8)	24 (37.5)			26 (47.3)	24 (43.6)		
Missing	8 (2.3)	4 (6.2)			1 (1.8)	3 (5.5)		

*AJCC* American Joint Committee on Cancer, *ALBI* Albumin-bilirubin, *PVTT* Portal vein tumor thrombosis, *SMD* Standardized mean difference

### Survival

**Fig. 2:**
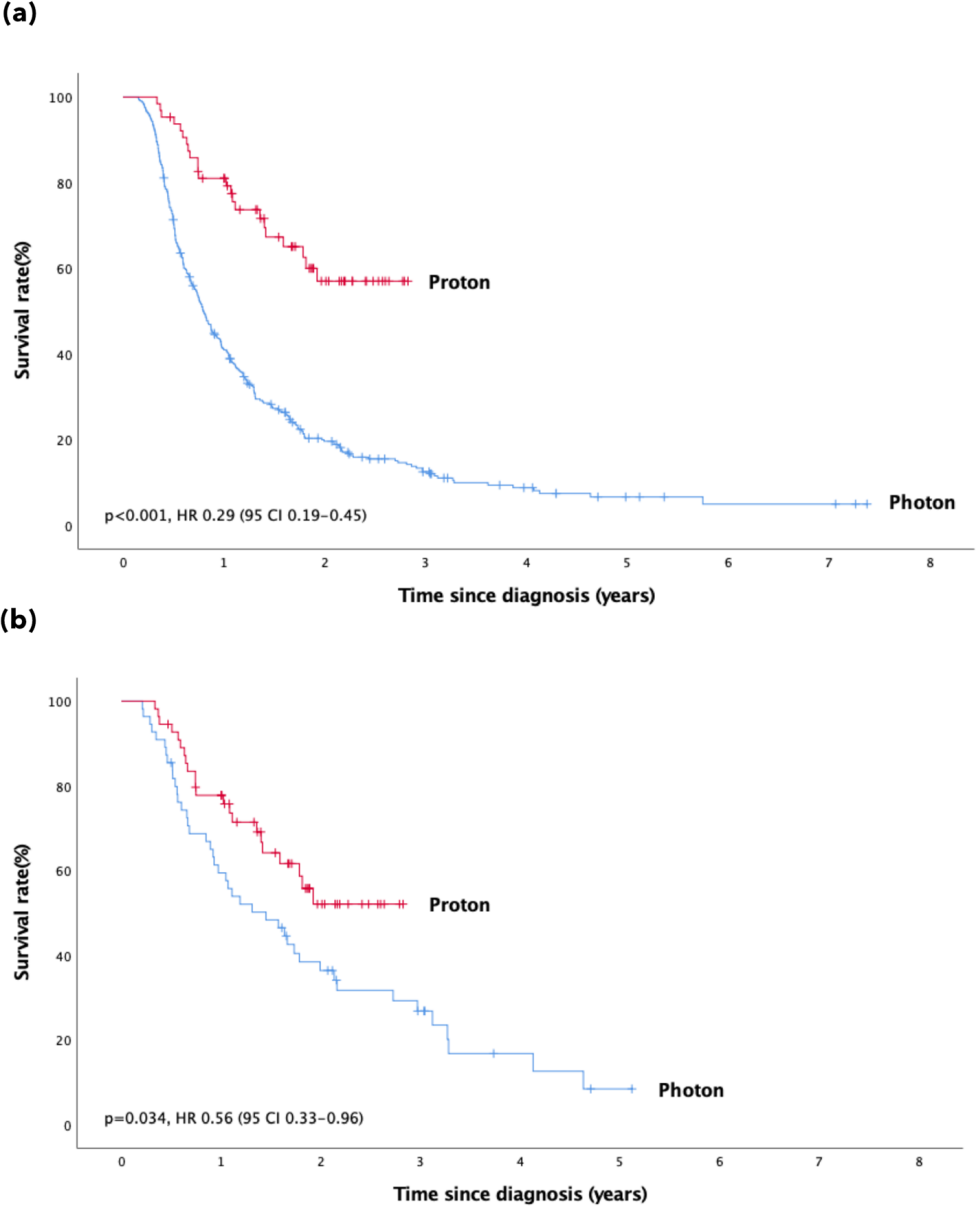
Overall survival before and after PSM. Legend: The overall survival rate with proton and photon radiotherapy before (**a**) and after PSM (**b**). PSM: propensity score matching

Cox regression analysis revealed a significant survival benefit in the proton group both before (*p* < 0.001, HR 0.29, 95% CI 0.19–0.45) and after PSM (*p* = 0.032, HR 0.56, 95% CI 0.33–0.96) (Fig. 2). The median OS in the photon group was 9.4 months and 17.4 months before and after PSM, respectively. The median OS in the proton group was not reached before or after matching.

**Fig. 3:**
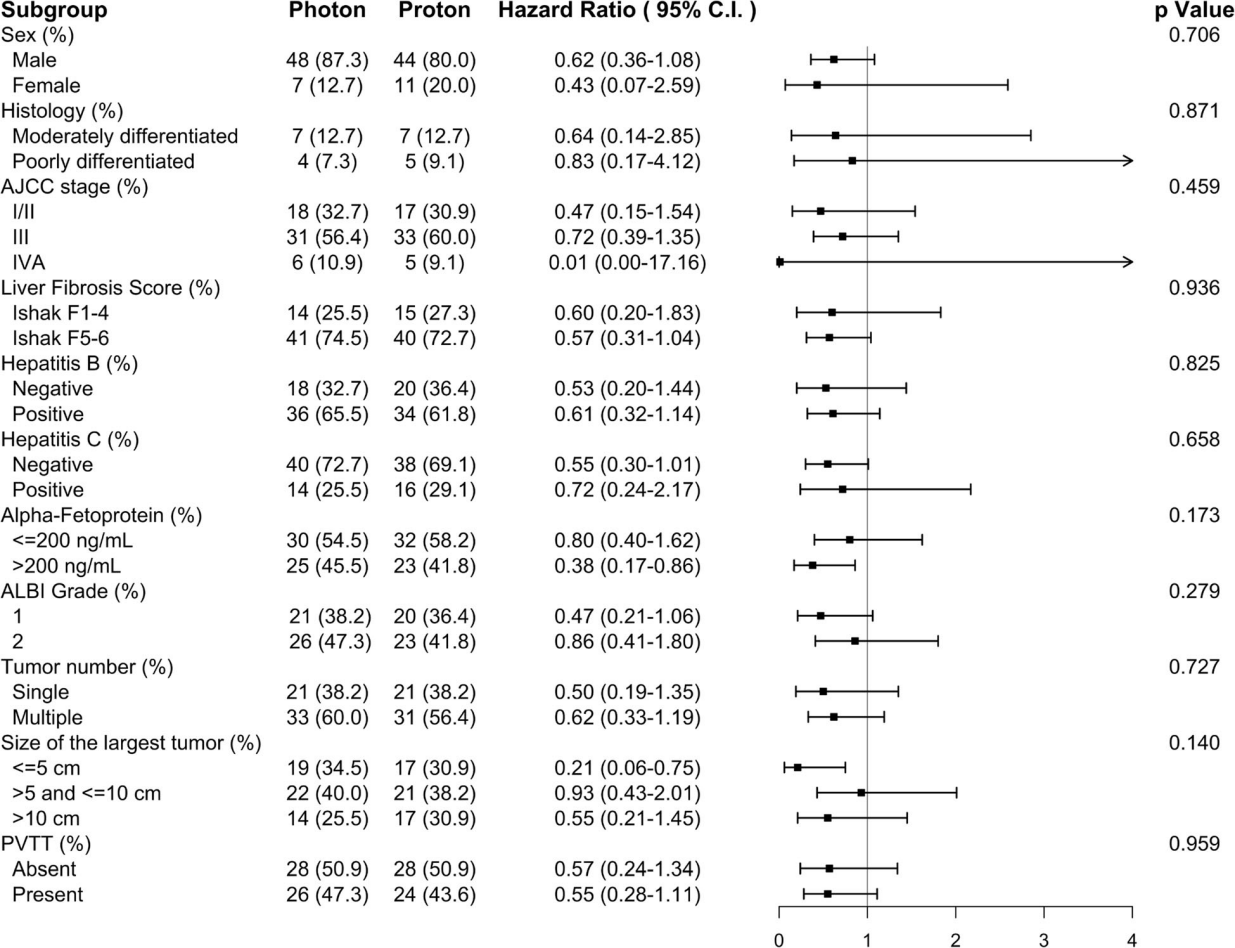
Stratified analyses of overall survival in the proton and photon groups. Legend: AJCC: American Joint Committee on Cancer; ALBI: albumin-bilirubin; PVTT: portal vein tumor thrombosis

Stratified analyses of OS in the matched cohort are presented in Fig. 3. The trend of the survival benefit of proton radiotherapy was consistent across all subgroups, and no significant heterogeneity in the HR was observed.

### BED and RILD

**Fig. 4:**
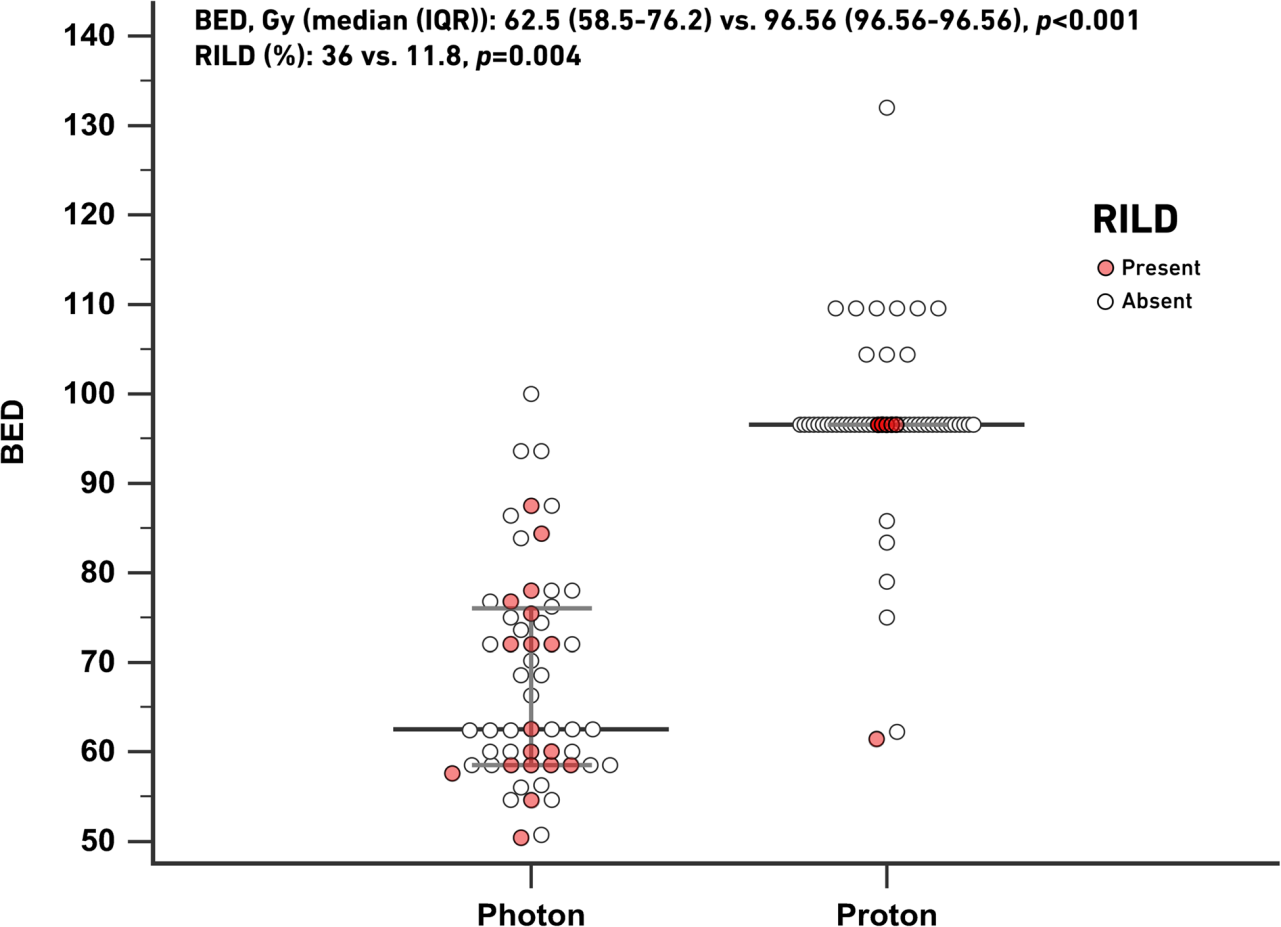
Distribution of the BED and cases of RILD. Legend: BED: biological equivalent dose; RILD: radiation-induced liver disease

The BED was significantly higher in the proton group than in the photon group (median, 96.56 (96.56–96.56) Gy (RBE) vs. 62.5 (58.5–76.2) Gy, *p* < 0.001). Among 110 patients in the PSM series, 101 patients had sufficient data available to evaluate RILD (photon: 50; proton: 51). There were 18 and 6 nonclassic RILD patients in the photon and proton groups, respectively. No classic RILD was recorded in either group. The risk of RILD was significantly lower in the proton group (11.8% vs. 36%, *p* = 0.004). The distribution of the BED and cases of RILD is presented in Fig. 4.

## Discussion

Clinical data comparing proton and photon radiotherapy with curative intent for HCC are limited. In the present study, we found that after appropriately adjusting for prognostic variables, patients who received proton radiotherapy had significantly better OS, which may be driven by a higher BED and lower risk of RILD.

These results echo those of the MGH report [8]. The study demonstrates survival benefits with proton therapy, which may be related to the decreased incidence of nonclassic RILD. It is always challenging for retrospective studies to minimize selection bias, especially when studying liver tumors, as both tumor characteristics and patients’ baseline liver function play a significant role in survival outcomes [11]. The strength of the present study is the balanced variables after PSM. We matched not only tumor variables but also baseline liver function variables that could potentially affect radiation toxicity and outcomes, including the liver fibrosis score, Child-Pugh class, ALBI grade, and hepatitis infection status [10, 12]. Moreover, we only included patients who underwent radiotherapy as the primary treatment modality after diagnosis, therefore precluding the effect of previous treatment modalities, such as radiofrequency ablation (RFA), surgery, or chemotherapy. The disease entity is also quite different between the present study and the MGH report. Our patients presented with more advanced HCC with larger tumors (median diameter of 6.8 cm for the largest tumor vs. median gross tumor volume of 106 ~ 118 mL) and a higher ratio of multiple tumors (56.4 ~ 60% vs. 36 ~ 49%), HBV infection (61.8 ~ 65.5% vs. 5 ~ 12%), and tumor thrombosis (43.6 ~ 47.3% PVTT vs. 27 ~ 35%), which may have influenced the BED that could be safely delivered by photon radiotherapy, which is much lower in the present study than in the MGH report (median, 62.5 Gy vs. 80.4 Gy [RBE]).

For liver tumors, it has been well demonstrated that a higher radiation dose could result in better oncological outcomes. The prescribed dose varies among series depending on the tumor entity, radiotherapy technique, and combined treatment modalities. In the era of 3D conformal radiotherapy (3DCRT), Park et al. analyzed 158 HCC patients and found that a higher radiation dose predicted a better tumor response rate (29.3% for < 40 Gy; 68.6% for 40–50 Gy, and 77.1% for > 50). However, the radiation dose also seemed to be a determining factor for RILD (4.2% for < 40 Gy; 5.9% for 40–50 Gy, and 8.4% for > 50 Gy) [3]. Among patients with PVTT who were treated with 3DCRT, Toya et al. reported a better response rate (80.0% vs. 21.7%, *p* < 0.001) and 1-year survival rate (59.3% vs. 29.2%, *p* = 0.04) for those who received BED ≥58 Gy [2]. In the era of intensity-modulated radiotherapy (IMRT), the radiation dose could be escalated safely by advanced techniques. Byun et al. analyzed 637 patients with Barcelona Clinic Liver Cancer (BCLC) stage C HCC who received IMRT with concurrent hepatic arterial 5-fluorouracil. A higher BED (≥72 Gy) significantly increased the 1-year local failure-free survival (95% vs. 78%; *p* = 0.008) and 1-year OS (62% vs. 51%; *p* = 0.03) rates [1]. Chadha et al. reported a series of 46 HCC patients treated with proton radiotherapy. Patients receiving a BED ≥90 Gy (RBE) had significantly better OS [7]. In the present study, the BED was significantly higher in the proton group and would be an essential factor contributing to better OS.

RILD is a major concern of using radiotherapy to treat liver tumors. The risk of RILD may be related to the liver radiation dose, the dose distribution, and underlying liver disease [13]. Investigations have tried to issue dose parameters to predict the risk of RILD [14]. In the Quantitative Analyses of Normal Tissue Effects in the Clinic (QUANTEC) report, Charlie et al. recommended that for those receiving therapeutic partial liver radiotherapy, keeping the mean normal liver dose (liver minus gross tumor volume) < 28 Gy for primary liver cancer and < 32 Gy for liver metastases may reduce the risk of RILD to < 5% [10]. Although advanced radiotherapy techniques such as IMRT or volumetric modulated arc therapy (VMAT) could achieve more conformal target dose coverage and a higher target dose than 3DCRT, the low-dose region may be increased by the nature of the physical properties of X-rays. The increase in the low-dose region in the normal liver could potentially increase the risk of RILD. Son et al. reported an analysis of 72 patients treated with helical tomotherapy and hypofractionated radiotherapy (40–50 Gy in 10 fractions). Normal liver receiving a dose of more than 15 Gy (V_15_) was found to be a parameter capable of predicting the deterioration of hepatic function [15]. Moreover, dosimetric studies have found that for liver tumors larger than 8 cm, IMRT or VMAT delivered a higher mean liver dose than 3DCRT [16]. These findings highlight the difficulties of using photon radiotherapy to treat large liver tumors optimally.

The story could be changed by the superior physical properties of proton radiotherapy. A proton beam has a finite range of energy deposition with no exit dose after the target. This physical advantage, compared with photon beams, may allow a higher target dose to be achieved without the unwanted spread of low doses to the surrounding normal liver. In a dosimetric study, Wang et al. demonstrated that for liver tumors, proton radiotherapy could significantly lower the mean liver dose and volume of normal liver receiving a dose of more than 30 Gy (RBE) (V_30_) compared to photon radiotherapy [5]. Toramatsu et al. also performed a dosimetric study to compare spot-scanning proton therapy (SSPT) and IMRT. They predicted the risk of RILD using the Lyman-normal-tissue complication probability model and found that the risk of RILD increased drastically between with IMRT but not SSPT for liver tumors 6.3–7.8 cm in diameter (RILD 94.5% for IMRT vs. 6.2% for SSPT for tumor size > 6.3 cm), which indicated that HCC lesions could be more safely treated with proton therapy, especially HCC lesions greater than 6.3 cm in size [17]. Moreover, patients with small normal liver volumes may potentially benefit from proton radiotherapy. Lee et al. analyzed 22 HCC patients with a small normal liver volume (< 800 cm^3^) who were treated with proton radiotherapy. The oncological results were a 1-year in-field local control rate of 95.5% and a 1-year OS rate of 81.8%; there were no cases of liver failure, and only one case of nonclassic RILD could be identified [18]. In the present study, 83.4% (*n* = 92) of the patients included in the PSM series had multiple tumors or a largest tumor size > 5 cm, and the risk of RILD would therefore be high with photon radiotherapy. Nonetheless, with proton radiotherapy, albeit with a higher BED, the risk of RILD was significantly lower, which is attributed to the advantages of its physical properties.

The dose schemas used in the proton cohort are mainly modified from Proton Medical Research Center (Tsukuba, Japan) protocols [19]. Briefly, 72.6 Gy (RBE) in 22 fractions or 66 Gy (RBE) in 10 fractions were prescribed, depending on the tumor location. The 72.6 Gy (RBE) protocol is preferred for tumors located within 2 cm of the gastrointestinal tract or porta hepatis. The physical advantage of the proton beam, which minimizes unwanted spreading of the dose to the surrounding normal liver, was that the prescribed doses in the proton cohort were quite uniform and mostly did not need to be tailored for large tumor volume HCC cases. On the contrary, in the photon cohort, large tumor volume may be an essential factor that resulted in higher unwanted doses spreading to the normal liver and therefore limited the tolerance of the prescribed dose. Consequently, photon dose prescriptions need to be tailored case by case for different tumor sizes or locations; therefore, the prescribed doses in the photon cohort were more diverse and lower than those in the proton cohort.

Our study does have potential limitations. Limited by the nature of the CGRD, we could only record the “size of the largest tumor” instead of the “total tumor volume.” Although the “size of the largest tumor” has been demonstrated to predict survival outcomes for HCC patients undergoing liver transplantation [20], this may result in uncertainties when assessing patients with multiple HCC lesions. The location of HCC is also an essential factor contributing to the dosimetric difference between photon and proton radiotherapy [21] and could not be addressed in the present study. Although we generated balanced groups according to several variables by PSM, potential selection bias is still present due to the retrospective nature of the study. Patients treated with proton radiotherapy in our series were all treated at a single medical center, while patients treated with photon radiotherapy were treated at four different hospitals. There may be a bias related to differences in protocols and treatment techniques among different hospitals. Economically, photon therapy is covered by National Health Insurance (NHI) in our country, while proton therapy is an expensive technique not covered by NHI. This may lead to potential selection bias regarding socioeconomic status between the groups.

## Conclusions

In this propensity-matched analysis, we demonstrated that compared to photon radiotherapy, proton radiotherapy could deliver a higher radiation dose without increasing the risk of RILD and result in a better overall survival rate for those diagnosed with HCC and treated with radiotherapy with curative intent. The physical advantages of proton therapy allow it to be used to treat HCC safely and potentially improve OS. Prospective investigations are needed to assess the role of proton radiotherapy in treating liver tumors.

## Data Availability

The datasets used or analyzed during the current study are available from the corresponding author on reasonable request.

## Abbreviations

OS: Overall survival
RILD: Radiation-induced liver disease
HCC: Hepatocellular carcinoma
MGH: Massachusetts General Hospital
ALBI: Albumin-bilirubin
CGRD: Chang Gung Research Database
IRB: Institutional review board
BED: Biological equivalent dose
RBE: Relative biological effectiveness
AJCC: American Joint Committee on Cancer
AFP: Alpha-fetoprotein
PVTT: Portal vein tumor thrombosis
PSM: Propensity score matching
SMD: Standardized mean difference
RFA: Radiofrequency ablation
3DCRT: 3D conformal radiotherapy
IMRT: Intensity-modulated radiotherapy
BCLC: Barcelona Clinic Liver Cancer
QUANTEC: Quantitative Analyses of Normal Tissue Effects in the Clinic
VMAT: Volumetric modulated arc therapy
SSPT: Spot-scanning proton therapy
NHI: National Health Insurance
